# Biofilm Formation of *Candida albicans* Facilitates Fungal Infiltration and Persister Cell Formation in Vaginal Candidiasis

**DOI:** 10.3389/fmicb.2020.01117

**Published:** 2020-06-05

**Authors:** Xueqing Wu, Sisi Zhang, Haiying Li, Laien Shen, Chenle Dong, Yao Sun, Huale Chen, Boyun Xu, Wenyi Zhuang, Margaret Deighton, Yue Qu

**Affiliations:** ^1^Department of Obstetrics and Gynecology, The First Affiliated Hospital of Wenzhou Medical University, Wenzhou, China; ^2^Department of Obstetrics and Gynecology, Shenzhen University General Hospital, Shenzhen, China; ^3^Department of Laboratory Medicine, The First Affiliated Hospital of Wenzhou Medical University, Wenzhou, China; ^4^School of Applied Sciences, RMIT University, Bundoora, VIC, Australia; ^5^Wenzhou Medical University-Monash BDI Alliance in Clinical and Experimental Biomedicine, Monash University, Clayton, VIC, Australia

**Keywords:** vaginal candidiasis, recurrent vaginal candidiasis, biofilm formation, fungal infiltration, persister cells, antifungal tolerance, mouse model

## Abstract

**Background:**

Vaginal candidiasis is an important medical condition awaiting more effective treatment. How *Candida albicans* causes this disease and survives antifungal treatment is not yet fully understood. This study aimed to establish a comprehensive understanding of biofilm-related defensive strategies that *C. albicans* uses to establish vaginal candidiasis and to survive antifungal treatment.

**Methods:**

A mouse model of vaginal candidiasis was adopted to examine the formation of biotic biofilms on the vaginal epithelium and fungal infiltration by laboratory and clinical strains of *C. albicans*. Histopathological changes and local inflammation in the vaginal epithelium caused by *C. albicans* of different biofilm phenotypes were compared. Antifungal susceptibility testing was carried out for *C. albicans* grown as planktonic cells, microplate-based abiotic biofilms, and epithelium-based biotic biofilms. Formation of persister cells by *C. albicans* in different growth modes was also quantified and compared.

**Results:**

*C. albicans* wild-type reference strains and clinical isolates, but not the biofilm-defective mutants, formed a significant number of biotic biofilms on the vaginal epithelium of mice and infiltrated the epithelium. Biofilm formation and epithelial invasion induced local inflammatory responses and histopathological changes in the vaginal epithelium including neutrophil infiltration and subcorneal microabscesses. Biofilm growth on the vaginal epithelium also led to high resistance to antifungal treatments and promoted the formation of antifungal-tolerant persister cells.

**Conclusion:**

This study comprehensively assessed biofilm-related microbial strategies that *C. albicans* uses in vaginal candidiasis and provided experimental evidence to support the important role of biofilm formation in the histopathogenesis of vaginal candidiasis and the recalcitrance of the infection to antifungal treatment.

## Introduction

Vaginal candidiasis is one of the commonest medical conditions affecting otherwise healthy women of reproductive age. It was estimated that 23–49% of women of reproductive age suffer from vaginal candidiasis, with the majority having uncomplicated infections (less than 3–4 episodes within a 12-month period) and a considerable number (6–9% of the patients) presenting with recurrent infections (at least 3–4 episodes in 1 year); the latter is a stubborn condition characterized by complex pathogenicity and tolerance to antifungal treatment (Ilkit and Guzel, 2011; Sobel, 2016; Blostein et al., 2017). Although vaginal candidiasis is believed to be an immunopathological condition of the human vagina with “neutrophy anergy” as the underlying mechanism, invading microorganisms might still play an important role in its pathogenicity (Yano et al., 2018). *Candida albicans* is the leading agent causing both uncomplicated and recurrent vaginal candidiasis (Liu et al., 2014; Goncalves et al., 2016). Proposed virulence determinants of *C. albicans* involved in the pathogenesis of vaginal candidiasis include fungal morphogenesis, adhesion to vaginal epithelial cells, the production of phospholipases and proteinase such as secreted aspartyl proteases (Saps), and the presence of candidalysin, a well-identified secreted cytolytic peptide toxin encoded by *ece1* (Kalkanci et al., 2013; Richardson et al., 2017).

While much has been done to define host- and pathogen-related factors contributing to the establishment of vaginal candidiasis (Ilkit and Guzel, 2011; Bruno et al., 2015; Yano et al., 2018), little effort has been placed on microbial mechanisms driving vaginal candidiasis to resist antifungal treatment and further develop into a more troublesome recurrent infection. Despite the fact that most *C. albicans* isolates from patients with uncomplicated or recurrent vaginal candidiasis showed sensitivities to many first-line antifungals, clinical ineffectiveness of these drugs in eradicating *Candida* or curing recurrent vaginal candidiasis has been constantly reported (Richter et al., 2005; Gamarra et al., 2014; Nagashima et al., 2016; Ying et al., 2016; Adjapong et al., 2017). This suggests that in addition to the host-related factors such as different host sensitivity to pathological responses, microbial strategies other than intrinsic resistance might be involved in the pathogenesis and persistence of vaginal candidiasis (Peters et al., 2014; Sobel, 2016).

*Candida albicans* and its related species are known to be able to adopt a major pathogenicity strategy, biofilm formation, to initiate and maintain infections, in particular on patients with breached immune defense (Hagerty et al., 2003; Kojic and Darouiche, 2004). Indeed, the role of epithelium-associated *C. albicans* biofilm in the development of vaginal candidiasis has been proposed (Harriott et al., 2010; Rodriguez-Cerdeira et al., 2019), though a recent study by Swidsinski et al. (2019) did not detect evident biofilms from the vaginal biopsies from patients with confirmed vaginal candidiasis. Two different types of biofilms might be involved in vaginal candidiasis: abiotic biofilms that require a plastic or metal substratum, such as intrauterine devices (IUDs; Auler et al., 2010; Cakiroglu et al., 2015), and biotic biofilms that use the vaginal epithelium as the supporting base (Harriott et al., 2010; Muzny and Schwebke, 2015). Epithelium-associated biotic biofilms were considered to be more important in the pathogenesis of recurrent vaginal candidiasis, as many patients suffer from recurrent infections without any IUDs implanted or after the removal of IUDs (Cakiroglu et al., 2015). Using a mouse vaginal candidiasis model, Harriott et al. (2010) successfully established *C. albicans* biofilms on the vaginal epithelium and raised the hypothesis that formation of epithelium-associated biofilms may be an initiating event for the infection (Harriott et al., 2010). A direct link between the formation of epithelium-associated biofilms and histopathological change of the vaginal epithelium, however, is still missing. Although antifungal resistance of *Candida* biofilms formed by clinical isolates from patients with vaginal candidiasis has been reported, the tests were mostly done on abiotic biofilms prepared in 96-well microplates (Gao et al., 2016; Sherry et al., 2017). Epithelium-associated biotic biofilms grown in a dynamic vaginal environment might differ from *in vitro* abiotic biofilms in many different ways, including their susceptibility to conventional antifungals (Bjarnsholt et al., 2013).

The typical dynamics of recurrent vaginal candidiasis coincides with the relapsing biofilm infection model proposed by Lewis (2010), suggesting possible involvement of persister cells (Lewis, 2010). Many studies, including our own, have found that persister cells are responsible for antimicrobial tolerance of many biofilm-related bacterial infections (Qu et al., 2010; Yang et al., 2015). Lafleur et al. (2006) first isolated *Candida* persister cells and linked their formation to the adherence growth mode of *C. albicans*. It is thus reasonable to hypothesize that biotic biofilms grown on the vaginal epithelium also harbor a small number of persister cells. Treatment of persister cells is known to be troublesome. Conventional antimicrobial agents, unless used at very high dose for an extended period, will not eradicate persister cells residing in biofilms (Yang et al., 2015; Wu et al., 2019). Our study aimed to establish a comprehensive understanding of the importance of *C. albicans* biofilm formation in the pathogenesis of vaginal candidiasis, in order to identify specific microbial targets for more efficient antifungal therapies.

## Materials and Methods

### Ethics Statement

This study was approved by the Ethics Committee of Wenzhou Medical University, China (Ethics approval number: Wydw2016-0214). All experiments were performed in accordance with the National Institutes of Health guide for the care and use of laboratory animals.

### Strains and Identification

Two biofilm-producing *C. albicans* laboratory reference strains, *C. albicans* DAY185 and *C. albicans* DAY286, and their biofilm-defective mutant strains, *med31*ΔΔ and *bcr1*ΔΔ, were used in this study (Lafleur et al., 2006; Uwamahoro et al., 2012). Two *C. albicans* clinical isolates designated as VVC2 and VVC4 were also included. These two isolates were from patients with clinically diagnosed vaginal candidiasis (two and four episodes within a 12-month period, respectively) at The First Affiliated Hospital of Wenzhou Medical University. The clinical isolates were identified to a species level using the following tests: CHROMagar *Candida* medium (CHROMagar, Paris, France) and the Vitek matrix-assisted laser desorption/ionization-time of flight mass spectrometry (MALDI-TOF MS, bioMérieux, Craponne, France).

### Mouse Vaginal Epithelial Biofilm Assay

An *in vivo* model of vaginal epithelium-associated biofilms was used essentially as previously described (Harriott et al., 2010). Firstly, BABL/C female mice of 6–8 weeks old were administered 0.1 mg of estrogen (17β-estradiol; Sigma) dissolved in 0.1 ml of sesame oil subcutaneously for three consecutive days prior to inoculation with *C. albicans. C. albicans* cells were grown in yeast peptone dextrose broth (YPD, 200 rpm, 30°C) for 20 h. One hundred microliters of fungal suspensions containing 7 × 10^5^ colony-forming units (CFU) of *C. albicans* cells was used to infect mice via an intravaginal pathway. Mice were maintained in an animal facility and euthanized after 72 h before the vagina was removed. Fungal growth on the vaginal epithelium and underlying tissue was assessed qualitatively using scanning electronic microscopy (SEM, see below) and quantitatively with the CFU-based viable count method.

### Scanning Electronic Microscopy

For SEM, dissected mouse vaginal tissues were fixed with glutaraldehyde for 2 h (2.5^%^, v/v, in 0.1 M cacodylate buffer, pH 7.0) at room temperature, and dehydrated with gradually increased ethanol levels (50, 75, 85, 95, 100, and absolute 100%) (Gargani et al., 1989). Samples were coated with gold using a Balzers SCD005 sputter coater and viewed under a scanning electron microscope (SEM; Hitachi, H-7500, Japan).

### CFU-Based Viable Counts

For CFU-based viable counts, dissected mouse vaginal tissues were weighted, homogenized in PBS using a tissue homogenizer, followed by vortexing vigorously for 30 s four times and sonication at 42 kHz for 10 min. The suspensions were serially diluted with PBS and plated on YPD for 72 h for enumeration of CFUs.

### Histopathology

For histopathological study of the infected vagina, tissues were fixed in 4% paraformaldehyde and selected tissue blocks were processed using a routine overnight cycle in a tissue processor. The tissue blocks were then embedded in wax, serially sliced into 5-μm sections. The transverse sections were stained with Periodic acid–Schiff (PAS) staining for the presence of *C. albicans* yeast/hyphae cells or Hematoxylin–Eosin (H&E) staining for tissue damage, and then imaged under a light microscope (Nikon ECLPSE 80i, Tokyo, Japan).

### Enzyme-Linked Immunosorbent Assay

Concentrations of two representative inflammatory effectors in mouse vaginal lavage fluids, innate cytokine IL-1β and an alarmin S100A8, were analyzed using commercially available enzyme-linked immunosorbent assay (ELISA) kits (Boyun Biotech Co., Ltd, Shanghai, China) per the instructions from the manufacturer (Peters et al., 2014). Enzyme immunoassay/radioimmunoassay (EIA/RIA) plates were incubated with dilutions of lavage fluid (at 1:10 for IL-1β and 1:100 for S100A8) and serially diluted protein standards for 2 h. After washing, the plates were treated with biotinylated polyclonal goat anti-mouse IL-1β or S100A8 antibodies for 2 h, followed by incubation with streptavidin horseradish peroxidase (HRP) for 20 min. A tetramethylbenzidine-H_2_O_2_ substrate solution was added to the plates and the reactions were measured with a microplate reader at 450 nm.

### *In vitro* Biofilm Formation on IUDs

*Candida albicans* biofilms were grown on two copper IUDs for qualitative analysis. Intrauterine devices were pretreated with fetal bovine serum (Sigma, North Ryde, Australia) overnight at 37°C with gentle shaking (75 rpm), washed twice with PBS, and transferred to a 24-well plate containing 1 ml of freshly prepared fungal suspensions (3 × 10^7^ cells/ml in RPMI-1640 at pH 4.0). The plate was incubated for 1.5 h at 37°C with gentle shaking (75 rpm) to allow the yeast cells to adhere to the surfaces. The IDUs were then gently washed with PBS and transferred to a new 24-well plate with RPMI-1640 media (pH 4.0), followed by incubation at 37°C with shaking (75 rpm) for 48 h. The established biofilms were examined with SEM. Two IUDs were used in this study; an indomethacin releasing round copper IUD (Medsuture, Shanghai, China), and a yuan-gong type copper IUD (Yantai Jishengyaoxie Co., Ltd., Shangdong, China).

### Antifungal Susceptibility Tests for Planktonic Cells and Abiotic Biofilms

Minimum inhibitory concentrations (MICs) were determined using the broth microdilution method according to CLSI guidelines M27-A3. Drug concentrations ranged from 0.125 to 64 μg/ml for clotrimazole and nystatin and 0.025–128 μg/ml for amphotericin B. The MIC was defined as the concentration resulting in complete growth inhibition for amphotericin B and an inhibition of at least 50% of fungal growth for other drugs, corresponding to a score of 2 in the NCCLS M27-A3 protocol. Abiotic biofilm MICs were determined by challenging *C. albicans* biofilms pre-grown in 96-well microplates with antifungal agents prepared in RPMI-1640 for 24 h (Qu et al., 2016). 3-Bis-(2-methoxy-4-nitro-5-sulfophenyl)-2H-tetrazolium-5-carboxanilide (XXT, Sigma-Aldrich, Australia) was used to assess the inhibitory efficacy of antifungal agents on the established abiotic biofilms. Biofilm MIC_80_ was determined as the lowest concentration that inhibited ≥80% of fungal biofilm growth. RPMI-1640 of pH 7.2 and pH 4.0 were used as growth medium, respectively, to examine the effect of different environmental acidity on antifungal efficacy as local pH has been found to impact on the antifungal-mediated killing of *C. albicans* (Ilkit and Guzel, 2011; Kasper et al., 2015).

### *Ex vivo* Antifungal Susceptibility Tests for Biotic Biofilms

For *ex vivo* antifungal susceptibility testing, epithelium-associated *C. albicans* biofilms were grown for 72 h before removal from the euthanized animals. Concentrations of selected antifungals ranged from 2 to 32 μg/ml for nystatin, 0.5–1280 μg/ml for clotrimazole, and 1–16 μg/ml for amphotericin B. Infected vaginal tissues were sectioned into small blocks of 3 mm × 5 mm × 5 mm and exposed to antifungal agents prepared at selected concentrations in RPMI-1640 (pH 4.0 and pH 7.2, respectively) for 24 h. The treated vaginal tissue blocks were washed with PBS, homogenized with tissue homogenizer. Colony-forming units counting was carried out by plating an aliquot onto YPD plates followed by an incubation at 37°C for 48 h. Vaginal tissue treated with RPMI-1640 served as untreated control.

### Quantification of Persister Cells From Planktonic Cultures, Abiotic Biofilms, and Biotic Biofilms

To isolate persister cells from planktonic cultures, suspensions of single cells from cultures at mid-log phase were adjusted to densities of ∼3 × 10^7^ CFU/ml and exposed to amphotericin B at 100 mg/ml for 24 h (Lafleur et al., 2006). To isolate the persister cell population from abiotic biofilms, *in vitro* biofilms of *C. albicans* were grown in 6-well microplates and collected using a cell scraper. Collected biofilms were then re-suspended into suspensions of single cells at densities of ∼3 × 10^7^ CFU/ml (LaFleur et al., 2010). Biofilm cells were statically exposed to amphotericin B at 100 mg/ml for 24 h. To isolate the persister cell population in epithelium-associated biotic biofilms, vagina tissues were removed from the euthanized animals and then sectioned into small blocks of 3 mm × 5 mm × 5 mm and homogenized with tissue homogenizer. Obtained suspensions were adjusted to densities of ∼3 × 10^7^ CFU/ml and statically challenged with amphotericin B at 100 mg/ml for 24 h. To avoid antifungal carryover and accurately quantify the number of persister cells, the antifungal treated suspensions were centrifuged at 3000 *g* for 5 min, washed twice with PBS, and re-suspended to the same volume with YPD broth. Colony-forming units counting was done by plating serially diluted aliquots onto YPD plates followed by incubation at 35°C for 72 h to maximize recovery of persister cells. The percentage of persister cells in different populations was calculated as follows: (fungal density after antifungal treatment)/(fungal density before antifungal treatment) × 100%, as described before (Wu et al., 2019).

### Data Analysis and Statistical Methods

One-way ANOVA or a non-parametric test was carried out to compare two means, depending on the data distribution. Statistical significance was assumed at a *p* value of less than 0.05. Data analysis was performed using Minitab 16 software (Minitab, State College PA, United States).

## Results

### *C. albicans* Forms Epithelium-Associated Biofilms in the Mouse Vagina Accompanied by Fungal Infiltration of the Epithelial Layer

**FIGURE 1:**
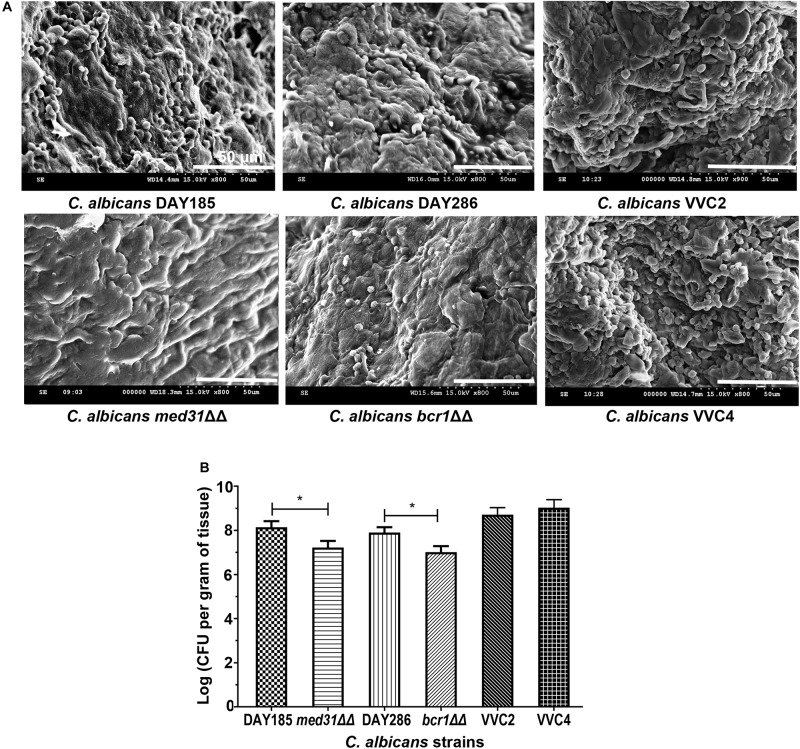
*Candida albicans* forms biotic biofilms on mouse vaginal mucosae. **(A)** Scanning electron microscopy of epithelium-associated biotic biofilms formed by *C. albicans* DAY185 and its *med31*ΔΔ mutant strain, *C. albicans* DAY286 and its *bcr1*ΔΔ mutant strain, and two clinical isolates from patients with vaginal candidiasis. The experiments were repeated on three different occasions. Scale bar = 50 μm. **(B)** Quantitative analysis of vaginal epithelium-associated biofilms formed by *C. albicans* DAY185 and its *med31*ΔΔ mutant strain, *C. albicans* DAY286 and its *bcr1*ΔΔ mutant strain, and two clinical isolates from patients with vaginal candidiasis. CFU-based viable counts were performed. The experiments were repeated three times in triplicate. Means and standard errors were presented. One-way ANOVA was used for two-set comparisons. *p < 0.05.

**FIGURE 2:**
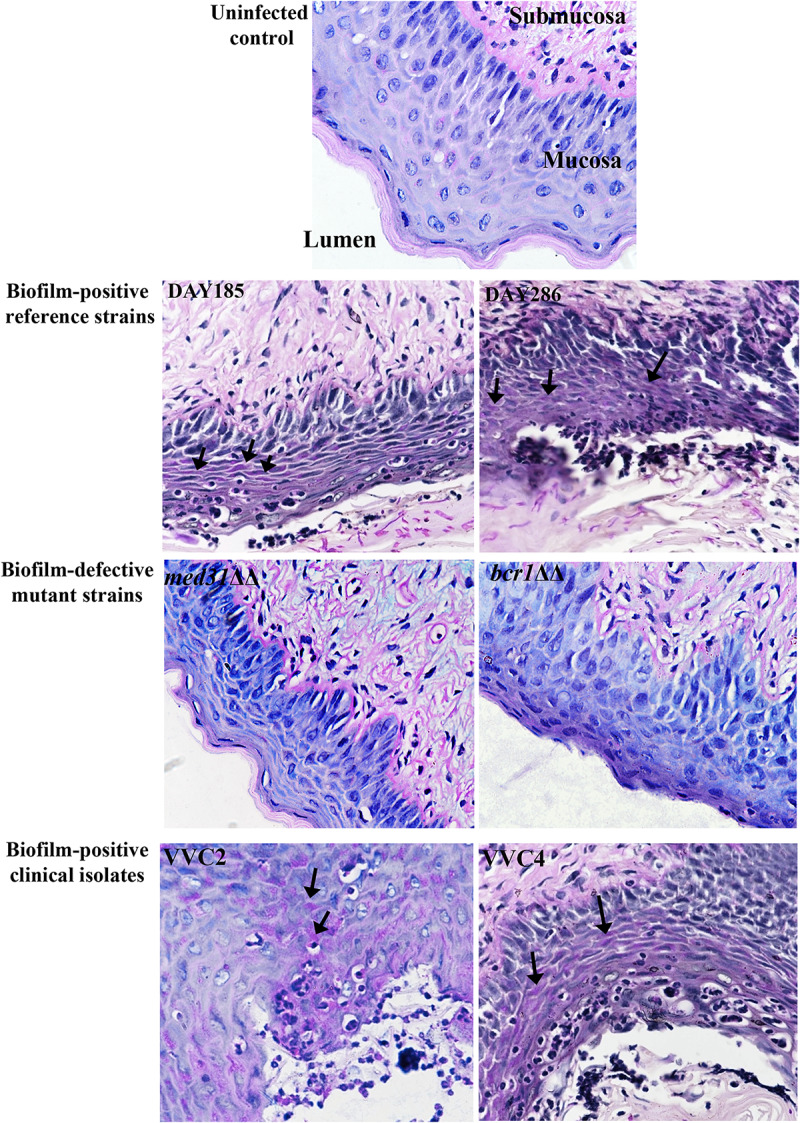
Histology of the vagina of mice infected by *C. albicans* shows fungal infiltration within the vaginal epithelium. Vaginal histopathological examination (PAS staining, 400×) showed that *C. albicans* penetrated the cornified epithelium and formed fungal infiltrations in the mucosal layer. The lumen, mucosa, and submucosa in the uninfected control are denoted for orientation. Evident endocytosed hyphae or fungal infiltrations (black arrows) in the mucosa were observed when *C. albicans* biofilm-positive reference strains (DAY185 and DAY286) and clinical isolates (VVC2 and VVC4), but not biofilm-negative mutants (*med31*ΔΔ and *bcr1*ΔΔ), were used to infect mice. The experiments were repeated on three different occasions.

**FIGURE 3:**
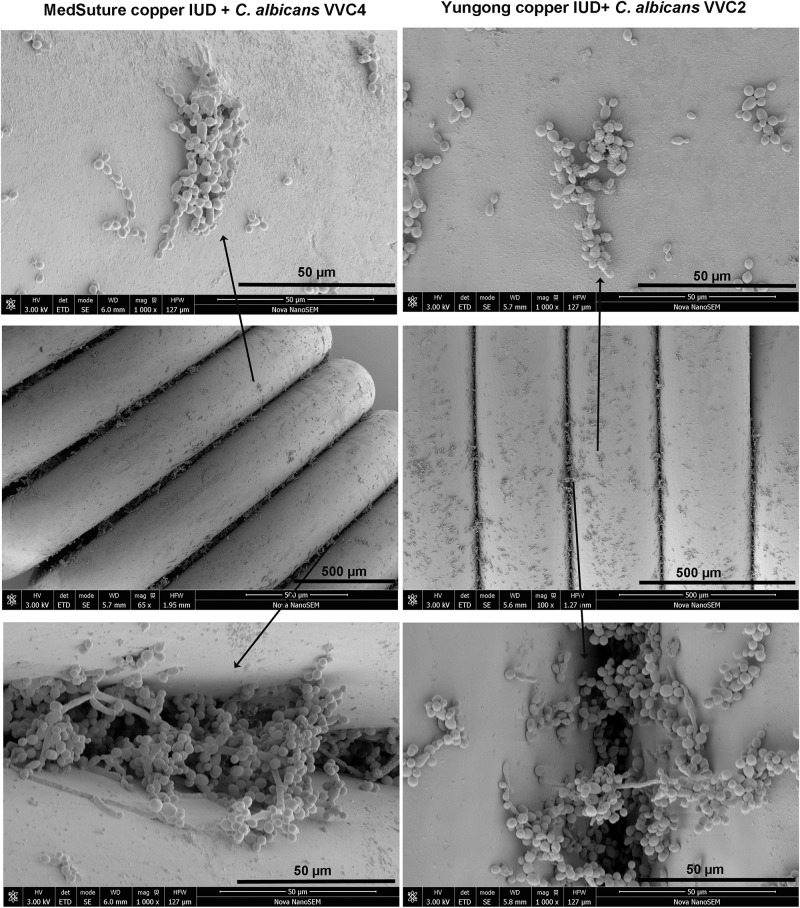
Biofilm formation on IUDs. Qualitative assessment of biofilm formation by *C. albicans* clinical isolates on IUDs. *C. albicans* biofilms were grown on two different IUDs for 48 h. Growth medium RPMI-1640 pre-adjusted to a pH of 4.0 was used for biofilm growth (as described in section “Materials and Methods”). SEM was employed for qualitative examination of biofilm formation. It was noticed that *C. albicans* formed three-dimensional biofilms on the IUD wire surface and in the inter-wire space of IUDs; the latter harbors more biofilm cells. The experiments were repeated on three different occasions.

We examined *C. albicans* laboratory strains and clinical isolates for *in vivo* biofilm formation on the mouse vaginal epithelium at 72 h post-inoculation. This time point was chosen because our preliminary experiment using *C. albicans* DAY185 suggested that fungal invasion and mouse immune response were both evident at this stage of the infection. Qualitative SEM showed that both DAY185 and DAY286 were able to irreversibly attach to the mouse vaginal epithelium, forming monolayer biofilms, with both yeast cells and hyphal elements distributed on the epithelium and apparently internalized by epithelial cells (Figure 1A). Two transcription factor mutant strains that are defective in biofilm formation *in vitro*, *med31*ΔΔ and *bcr1*ΔΔ, also formed monolayer biofilms on the vaginal epithelium, but to a lower extent (Figure 1A). Parallel quantitative viable counts also suggested biofilm formation of *C. albicans* on the vaginal epithelium, with *med31*ΔΔ and *bcr1*ΔΔ showing densities approximately 1 log CFU/g of infected tissue less than that of the wild-type strains (Figure 1B). Periodic acid–Schiff staining clearly showed *Candida* hyphal infiltration of the vaginal epithelial layers. This phenotype, however, was only found for wild-type DAY185 and DAY286, not the mutant strains (Figure 2). Two clinical isolates from patients with vaginal candidiasis also formed significant biofilms on the mouse vaginal epithelium and presented epithelial infiltration, as suggested by SEM imaging, viable counts, and histopathological examination (Figures 1A,B, 2). We also assessed biofilm formation of *C. albicans* clinical isolates on IUDs, using an *in vitro* model of biofilm cultivation. At a vaginal physiological pH of 4.0, *C. albicans* clinical isolates VVC2 and VVC4 formed three-dimensional multilayer biofilms on the surface of IUD wire and in the gaps between neighboring wires (Figure 3). The intricate three-dimensional structure of the IUDs, particularly the inter-wire space, appeared to support robust biofilm formation of *C. albicans* (Figure 3).

### Histopathological Changes of the Vaginal Epithelium Responsive to the Formation of Biotic *C. albicans* Biofilms and Fungal Infiltration

**FIGURE 4:**
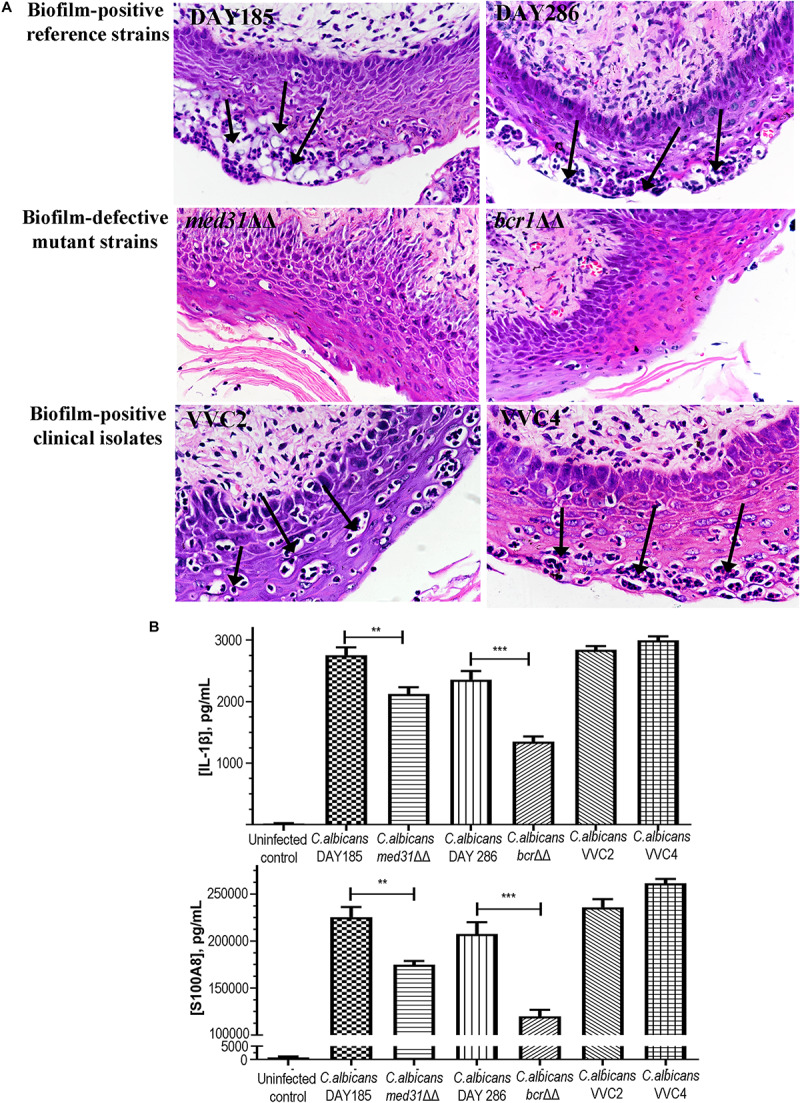
Histopathological and inflammatory changes of the vaginal epithelium after exposure to *C. albicans* of different biofilm phenotypes. **(A)** Vaginal histopathological examination (H&E staining, 400×) showed the formation of numerous microabscesses and neutrophil infiltration (black arrows) in the cornified epithelium after mice were infected with biofilm-positive *C. albicans* reference strains (DAY185 and DAY286) and clinical isolates (VVC2 and VVC4). Minimal neutrophil infiltration and microabscesses were seen when biofilm-defective mutant strains (*med31*ΔΔ and *bcr1*ΔΔ) were used to infect the mice. The experiments were repeated on three different occasions. **(B)** Local inflammatory responses to *C. albicans* infections in the mouse vagina. *C. albicans* DAY185 and its *med31*ΔΔ mutant strain, *C. albicans* DAY286 and its *bcr1*ΔΔ mutant strain, and two clinical isolates from patients with vaginal candidiasis were tested respectively. Cytoplasmic protein S100A8 and cytokine IL-1β were selected as the representative inflammatory effectors. The experiments were repeated three times in duplicate. Means and standard errors were shown. One-way ANOVA or a non-parametric test was used for two-set comparisons. **p < 0.01, ***p < 0.001.

One of the key research questions, and also the focus of this study was whether the presence of epithelium-associated biofilms is directly related to the pathological changes within vaginal tissues. We assessed the histopathological changes of the vaginal epithelium and associated alarmin/cytokine responses. Hematoxylin–Eosin staining showed evident neutrophil infiltration and subcorneal microabscesses, two changes of vaginal mucosae typically related to fungal infections in the epithelial layer (Figure 4A). Little change was found when mutant strains (*med31*ΔΔ and *bcr1*ΔΔ) were used to infect the mice (Figure 4A). Enzyme-linked immunosorbent assay targeting cytoplasmic protein S100A8 and cytokine IL-1β demonstrated a raised level of both agents in mice infected with any of the *C. albicans* strains and indicated an inflammatory condition (Figure 4B). *C. albicans* DAY185 and DAY286 had readings of S100A8 and IL-1β significantly higher than the mutant strains (*med31*ΔΔ and *bcr1*ΔΔ), suggesting a possible linkage between the formation of biotic biofilms by *C. albicans*, fungal infiltration, and the harmful immune responses to infected vaginal tissues. Significant histopathological changes and induction of inflammatory responses were also observed when two clinical isolates were introduced to the mouse vagina (Figure 4B).

### Formation of Epithelium-Associated Biofilms by *C. albicans* in the Vagina Leads to Higher Antifungal Resistance

We further tested antifungal susceptibilities of DAY185 and two clinical isolates grown in different modes, including planktonic cells, microplate-based abiotic biofilms, and epithelium-associated biotic biofilms, to three conventional agents under neutral and acidic conditions, respectively (Table 1). At pH 7.2, planktonic cultures of DAY185 and both clinical isolates remained sensitive to all three agents used in this study. Acidic condition of the vagina significantly impacted on the susceptibility of nystatin and clotrimazole but not amphotericin B, increasing MICs of nystatin and clotrimazole by 4–16 times. When grown as abiotic biofilms, *C. albicans* strains generally gained higher resistance to clotrimazole (ratio biofilm MIC80/planktonic MIC: >160-fold) and nystatin (1- to 16-fold) and remained relatively sensitive to amphotericin B (1- to 4-fold). Epithelium-associated biotic biofilms demonstrated highly elevated resistance to antifungal agents, with two clinical isolates showing biofilm MIC80 even higher than that of microplate-based abiotic biofilms (Table 1).

**TABLE 1:** Antifungal susceptibility of *C. albicans*: planktonic cells, microplate-based abiotic biofilms, and vaginal epithelium-associated biotic biofilms.

Antifungals	Planktonic MIC (mg/l)			Abiotic biofilm MIC80 (mg/l)			Biotic biofilm MIC80 (mg/l)		
	*C. albicans* strains			*C. albicans* strains			*C. albicans* strains		
	DAY185	VVC2	VVC4	DAY185	VVC2	VVC4	DAY185	VVC2	VVC4
**pH 7.2**									
Nystatin	4	2	4	32	64	64	32	>32	>32
Clotrimazole	0.5	2	1	>1280	>1280	>1280	1280	1280	>1280
Amphotericin B	1	2	1	1	4	4	2	16	16
**pH 4.0**									
Nystatin	16	32	32	16	64	128	32	32	>32
Clotrimazole	8	8	16	1280	>1280	>1280	>1280	>1280	>1280
Amphotericin B	2	2	4	2	4	16	2	>16	>16

*Experiments for antifungal susceptibility testing were repeated on three different occasions in triplicate. Geometric means of planktonic MICs and biofilm MIC80s were calculated and presented in the table. Definitions and detailed methods for planktonic MIC and biofilm MIC80 can be found in the main text.*

### Formation of Epithelium-Associated Biotic Biofilms by *C. albicans* Prompts the Formation of Persister Cells

**FIGURE 5:**
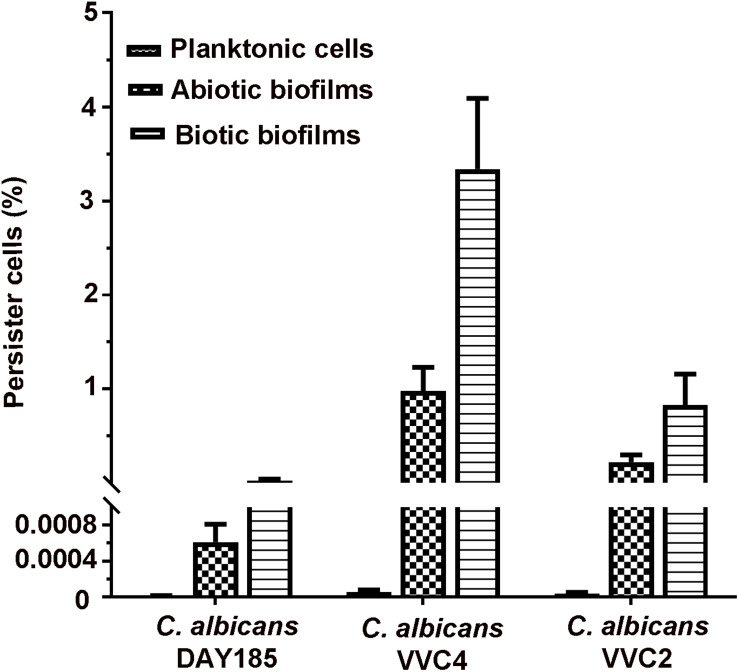
Quantitative assessment of persister cells formed by *C. albicans* in three different growth modes. *C. albicans* cells were grown as planktonic cells, abiotic biofilms on tissue culture-treated polystyrene surfaces, and biotic biofilms on the mouse vaginal epithelium, respectively. Biofilm cells and planktonic cells were re-suspended to a density of ∼3 × 10^7^ CFU/ml in RPMI-1640, followed by exposure to amphotericin B at 100 mg/l overnight. Survivors were recovered after 72 h incubation on YPD plates and counted as the percentage of the initial population. The experiments were repeated three times in triplicate. Means and standard errors were presented.

Persister cells residing in planktonic cultures, microplate-based abiotic biofilms, and epithelium-associated biotic biofilms were quantified. When grown as planktonic cultures at a mid-log phase, *C. albicans* DAY185 and two clinical isolates produced only very few persister cells (0.0001–0.0008%; Figure 5). Formation of abiotic biofilms by *C. albicans* on biomaterials significantly increased the number of persister cells in the population; the percentage seemed to be strain-dependent with DAY185 reaching 0.05%, VVC2 reaching 0.1%, and VVC4 reaching 1%. Growth of biotic biofilms on the vaginal epithelium further promoted persister cells of VVC2 to 0.6%, and VVC4 to 3.3%, significantly higher than that for abiotic biofilms or planktonic cultures (Figure 5).

## Discussion

Biofilm formation has been proposed as one of the most important virulence factors of *C. albicans* causing vaginal candidiasis, contributing to the establishment and recurrence of the infection (Muzny and Schwebke, 2015). Although biofilm formation of *C. albicans* has been extensively studied with clinical isolates from vaginal candidiasis patients, most studies used *in vitro* systems for biofilm cultivation that might only represent biofilms formed on IUDs (Cakiroglu et al., 2015; Gao et al., 2016; Sherry et al., 2017). Only a few studies have been carried out on biofilms grown on the vaginal epithelium (Harriott et al., 2010; Peters et al., 2014). Harriott et al. (2010) characterized *in vivo* biofilm formation of *C. albicans* on the vaginal epithelium; Peters et al. (2014) discovered a link between fungal morphogenesis of *C. albicans* and the immunopathology of vaginal candidiasis (Peters et al., 2014). Using the mouse model developed by Harriott et al. (2010), we further dissected the role of *C. albicans* biofilms in the histopathogenesis and persistence of vaginal candidiasis. Key findings of our study include the following: *C. albicans* infects the vagina by establishing epithelium-associated biofilms and fungal infiltration; histopathological changes of the vaginal epithelium and local inflammation are in relation to the formation of epithelium-associated biofilms; biofilm formation by *C. albicans* on the vaginal epithelium leads to high antifungal resistance; formation of biotic biofilms on the vaginal epithelium promotes the formation of persister cells.

By comparing biofilm-positive *C. albicans* reference strains and their biofilm-defective mutant strains, we established a link between biofilm formation and the disease state of vaginal candidiasis. *C. albicans* wild-type reference strain DAY286 and its *bcr1*ΔΔ mutant have been previously studied for *in vivo* biofilm formation on the mouse vaginal epithelium (Nobile et al., 2006; Harriott et al., 2010). Bcr1 is a transcription factor regulating many important biological processes in *C. albicans*; its mutant demonstrates normal filamentation but a reduction in biofilm formation on abiotic substrate and host organs (Nobile et al., 2006). Another wild-type reference strain *C. albicans* DAY185 experimentally causes systematic infection in mice; its *med31* deletion mutant showed a reduction in both filamentation and biofilm formation (Uwamahoro et al., 2012). Our study found that biofilm-positive wild-type reference strains, not the biofilm-defective mutants, induced evident histopathological damages and local inflammation, supporting an important role of biofilm formation in the histopathogenesis of vaginal candidiasis. One hurdle in attributing biofilm formation of *C. albicans* to its pathogenesis is to exclude other co-existing virulence strategies that might also be involved in *C. albicans* pathogenicity (Sudbery, 2011). Most transcription factors or their encoding genes often synchronously regulate biofilm formation of *C. albicans* and other important programs such as yeast-hyphae morphogenesis (Nobile et al., 2006; Sudbery, 2011; Uwamahoro et al., 2012). The pathogenesis of vaginal candidiasis has been essentially linked to the hyphal invasive form of *C. albicans* that is required to penetrate the intact epithelial layer and induce immunopathology (Sudbery, 2011). Fortunately, the *bcr1*ΔΔ mutant, which has a defect in biofilm formation but not morphogenesis (Peters et al., 2014), demonstrated a reduced capacity to elicit an immune response or histopathological damage, as found in this study. We also noticed a relation between biofilm formation and candida infiltration of the vaginal epithelium, with biofilm-defective mutant strains presenting less fungal infiltration. Recent work by Swidsinski et al. (2019) found endocytosed hyphae or pseudohyphae in the vaginal epithelium in patients with confirmed vaginal candidiasis, although no typical multi-layer biofilm structure was detected in the human vagina (Swidsinski et al., 2019). We propose that the biofilm-related fungal infiltration might resemble microcolony biofilms that are more frequently found *in vivo*, and share some important traits with *in vivo* biofilms, such as harboring antifungal-tolerant persister cells (Bjarnsholt et al., 2013; Xu et al., 2019). It is now known that dense microbial growth in a confined space promotes the formation of persister cells (Qu et al., 2010).

We also presented experimental evidence to explain the tolerance of vaginal candidiasis to antifungal treatment. Similar to microplate-based abiotic biofilms, epithelium-associated biotic biofilms are highly resistant to first-line antifungals. More importantly, epithelium-associated biofilms act as a conducive environment that facilitates the formation of persister cells. Using a regimen that kills the bulk of fungal cells with normal susceptibilities, our recent study has successfully isolated persister cells from the vaginal epithelium of mice infected with *C. albicans* (Wu et al., 2019). These persister cells could not be eradicated by conventional antifungal drugs at very high concentrations and might be the main culprit responsible for the recalcitrance of infections to antifungal treatment (Wu et al., 2019). The current study further established a connection between biofilm growth by *C. albicans* in the vaginal epithelium and the formation of persister cells, supporting a role of *Candida* biofilm formation in the recurrence of vaginal candidiasis.

One of the possible limitations of this study was that the mouse model of vaginal candidiasis still differs from human vaginal candidiasis in several physical aspects, including a lack of *C. albicans* as part of the vaginal microbiota, neutral vaginal pH, and dependence on exogenous estrogen to initiate fungal colonization (Harriott et al., 2010; Cassone and Sobel, 2016). Differences in responses to *Candida* infection between human and mice or other rodents cannot be neglected (Sobel, 2015; Vecchiarelli et al., 2015; Cassone and Sobel, 2016). This small animal model, however, remains a valuable research tool to study vaginal candidiasis as it closely parallels the chronic nature of the diseases in women and is cost-effective (Vecchiarelli et al., 2015; Yano et al., 2018). Another limitation is the small number of clinical isolates used in this study. The conclusion drawn from this study might not fully represent vaginal candidiasis caused by other *C. albicans* clinical isolates or other *Candida* spp. The cost, labor, and requirements from the ethics aspect have limited the use of a greater number of *C. albicans* strains in our study.

## Conclusion

In summary, our study provides a comprehensive understanding of biofilm-related factors involved in the pathogenicity and persistence of vaginal candidiasis caused by *C. albicans.*

## Data Availability Statement

The datasets generated for this study are available on request to the corresponding author.

## Ethics Statement

The animal study was reviewed and approved by Ethics Committee of Wenzhou Medical University, China.

## Author Contributions

YQ and XW conceived and designed the study. SZ, HL, CD, LS, YS, HC, BX, WZ, and YQ carried out the experiments. YQ, SZ, XW, and MD performed data analysis. YQ wrote the manuscript. YQ, MD, and XW edited the manuscript. All authors reviewed the manuscript and provided critical comments.

## Conflict of Interest

The authors declare that the research was conducted in the absence of any commercial or financial relationships that could be construed as a potential conflict of interest.
